# Circulating Endothelial Cells and Microparticles as Prognostic Markers in Advanced Non-Small Cell Lung Cancer

**DOI:** 10.1371/journal.pone.0047365

**Published:** 2012-10-15

**Authors:** Tania Fleitas, Vicenta Martínez-Sales, Virtudes Vila, Edelmiro Reganon, David Mesado, Maria Martín, José Gómez-Codina, Joaquín Montalar, Gaspar Reynés

**Affiliations:** 1 Department of Medical Oncology, Hospital Universitario y Politécnico La Fe Valencia, Valencia, Spain; 2 Research Center,Hospital Universitario y Politécnico La Fe Valencia, Valencia, Spain; Ospedale Pediatrico Bambino Gesu’, Italy

## Abstract

**Background:**

Circulating endothelial cells and microparticles have prognostic value in cancer, and might be predictors of response to chemotherapy and antiangiogenic treatments. We have investigated the prognostic value of circulating endothelial cells and microparticles in patients treated for advanced non-small cell lung cancer.

**Methodology/Principal Findings:**

Peripheral blood samples were obtained from 60 patients before first line, platinum-based chemotherapy +/− bevacizumab, and after the third cycle of treatment. Blood samples from 60 healthy volunteers were also obtained as controls. Circulating endothelial cells were measured by an immunomagnetic technique and immunofluorescence microscopy. Phosphatidylserine-positive microparticles were evaluated by flow cytometry. Microparticle-mediated procoagulant activity was measured by the endogen thrombin generation assay. Results: pre- and posttreatment levels of markers were higher in patients than in controls (p<0.0001). Elevated levels of microparticles were associated with longer survival. Elevated pretreatment levels of circulating endothelial cells were associated with shorter survival.

**Conclusions/Significance:**

Circulating levels of microparticles and circulating endothelial cells correlate with prognosis, and could be useful as prognostic markers in patients with advanced non-small cell lung cancer.

## Introduction

Lung cancer is one of the malignant tumors with the highest incidence worldwide. Non-small-cell lung cancer (NSCLC) represents more than 80% of lung cancer cases, and most of them have advanced or metastatic disease at diagnosis [1].

Platinum-based chemotherapy is the standard treatment for advanced lung cancer. Several platinum doublets are currently used with similar efficacy, though different toxicity profiles [2]. Progress in the understanding of cancer biology have driven the development of drugs against specific molecular targets, such as epidermal growth factor receptor (EGFR) and vascular endothelial growth factor (VEGF) and its receptors (VEGFRs). Some cytotoxic agents and targeted drugs are safer or more effective in specific histological subtypes, thus allowing more personalized treatments [3]. Bevacizumab, a humanized antibody against VEGF, has demonstrated a modest increase in progression-free survival (PFS) and overall survival (OS) in NSCLC when combined with chemotherapy. However, its use is restricted to patients with non-squamous cell histology [4]. Despite these advances, only a fraction of patients benefit from treatment and most of them die within 2 years after diagnosis. Hence, there is a need for biomarkers that allow identifying patients who are more likely to respond to treatment.

Circulating endothelial cells (CECs) are markers of vascular damage [5] that may have clinical relevance in cancer [6]–[7]. CEC count is increased in cancer patients [8]–[9] and correlates with tumor progression [10]. It has been suggested that the quantification of CECs is useful for identifying patients who might benefit from angiogenesis inhibitors and for monitoring treatment response [11]. In advanced colorectal cancer patients, a high baseline CEC count is associated with a shorter median PFS [12]–[13]. However, in patients with breast cancer and with NSCLC, a high baseline CEC count is associated with a prolonged PFS [11], [14].

Microparticles (MPs) are small vesicles (100 nm–1 µm) which directly bud from the plasma membrane [15]–[16]. They are released by different cells, including blood, endothelial and tumour cells [16]–[17]. The exposure of phosphatidylserine on the outer surface of the membrane is a specific characteristic of MPs [18]; their presence significantly increases the procoagulant activity of MPs by promoting the assembly of components of the clotting cascade. Furthermore, tumor cells generate an elevation of MPs that express tissue factor [19]–[21], which confers them a procoagulant potential [22]. In this regard, it has been reported that levels of circulating MPs are correlated with endogen thrombin generation (ETG) [23]. MPs are increased in cancer patients and they play a role in cancer related hypercoagulability [20]–[21], [24]–[26]. In addition, the emission of microvesicles, such as exosomes and MPs, has important pathological roles in tumor progression, facilitating the release and spread of cancer cells to generate metastases [27]. MPs retrieved from the peripheral blood of cancer patients may provide relevant information regarding prognosis and treatment response.

The aim of this study was to assess CECs, MPs and MP-mediated procoagulant activity in patients with advanced NSCLC, before and after standard chemotherapy, to analyze their prognostic value and to establish their possible association with tumor response.

## Materials and Methods

### Study Design and Patients

This prospective study included consecutive patients with NSCLC treated at the Medical Oncology Department of La Fe University Hospital with first-line chemotherapy of platinum-based doublets. Bevacizumab was added to treatment when indicated. To be included in the study, patients were required to meet the following criteria: age >18 years; stage IIIB or stage IV histologically confirmed NSCLC; life expectancy ≥12 weeks; ECOG Performance Status (PS) ≤2 and adequate renal, hepatic, and bone marrow function.

Staging was determined by chest and abdominal computed tomography (CT) and body proton emission tomography (PET) scanning. Three weeks after the third cycle of treatment, CT-based clinical assessment was carried out using the Response Evaluation Criteria for Solid Tumors (RECIST) [28]. After treatment, patients were assessed every three months. Blood biomarkers were evaluated at baseline and three weeks after the third cycle of treatment. The control group was composed of healthy subjects matched in sex and age with patients. Patients and controls provided written informed consent. This study was approved by the institutional Biomedical Research Ethics Committee.

### Biomarker Evaluation

Blood samples were obtained by cubital non-traumatic vein puncture, and collected into Vacutainer ™ plastic tubes (BD Diagnostic) with two different anticoagulants: K3 EDTA (4.3 mg) for CECs, and sodium citrate (129 mM) at a ratio of 1∶9 (v/v, sodium citrate/blood) for MPs and ETG. Samples were kept at 4°C and processed between 2 and 4 hours after collection. Citrated blood samples were centrifuged at 1,500 xg for 30 minutes at 4°C to obtain platelet-free plasma, and then stored at −80°C to allow later batch analysis.

### Quantification of Total Microparticles

Plasma samples containing MP suspensions were analyzed by flow cytometry. Annexin V-FITC conjugates were used to detect accessible phosphatidylserine on MP membranes. Ten µL of frozen plasma samples were diluted in 500 µL of HEPES buffer containing 2.5 mM CaCl_2_ (pH 7.4). Samples were then incubated for 60 minutes at 4°C in darkness with 1 µL of FITC-Annexin V (TACS AnnexinV, Trevigen Inc). The acquisition of events was quantified in an EPICS XL- cytometer (Beckman Coulter) at high flow rate for 2 minutes during which 325 µL of this suspension were analyzed. Standard fluorescent beads of different diameters were used for size calibration (0.5–3.0 microns, Megamix, BioCytex) and to set the gate of MP detection at a diameter from 0.5 to 1 µm, following a consensus guideline on MP measurement [29]. The size of MPs correlated with forward scatter (FS) and their granularity with side scatter (SS) parameters. The read-channels FS, SS and fluorescence were set at logarithmic gain. To limit background noise from dust and crystals, all dilution buffers were filtered using 0.22 µm filters (Millipore). To differentiate MPs from events due to noise, MPs were identified as particles >0.5 and <1.0 mm in diameter, and positively stained for Annexin V. MP suspension in plasma was prepared without calcium as a negative control. The number of FITC-Annexin V positive MPs was calculated and expressed as events per µL of plasma.

### Thrombin Generation Measurements

ETG assays were carried out to assess the in vitro thrombin generating capacity of plasma by using the calibrated automated thrombogram (CAT, Thrombinoscope BV). Eighty µL of frozen plasma samples were dispensed into 96-well round-bottom microtiter plates (Greiner Bio-One, ref650061, USA). Each sample requires two wells. Twenty µL of standard thrombin calibrator (TS20.00 Thrombin Calibrator, Thrombinoscope BV) were added to one well, and 20 µL of fluorogenic thrombin substrate with calcium (TS50.00 Fluka Kit Thrombinoscope BV) were added to both wells to start ETG with continuous comparison to the thrombin calibrator reagent. Thrombin activity was measured as fluorescence in a Fluoroskan Ascent reader fluorimeter (Thermo Labsystems) equipped with a 390/460 filter set. No exogenous tissue factor or phospholipids were added. Thrombin generation curves were calculated using the Thrombinoscope software and results were expressed as thrombin peak (nM) (height of the curve).

### Quantification of Circulating Endothelial Cells

The isolation and quantification of CECs were performed in whole blood by an immunomagnetic technique, following a consensus protocol [30]. CECs were isolated from whole blood at 4°C with PanMouse M450 Dynabeads (Dynal, Oslo, Norway) coated with sEndo1 (Biocytex, Marseille, France), a monoclonal antibody raised against the endothelial antigen CD146. To avoid non-specific binding of leukocytes to CD146 coated beads, cells were incubated after immunomagnetic isolation with FITC-labeled Ulex Europaeus Lectin-1, (UEA1) (Sigma ALDRICH, Inc) for 1 h in darkness, thus confirming the endothelial nature of the isolated cells. Samples were washed and suspended in buffer, and cells counted under fluorescence microscopy using a Nageotte chamber. Nucleated cells >10 µm in length, with more than five immunomagnetic beads attached and positive UEA1 staining were considered as CECs. Conglomerates were counted as one cell. The number of CECs was expressed as cells per mL of blood. Reproducibility was tested by performing six replicates of ten different samples.

### Statistical Analysis

Results are given as mean values ± standard deviations (SD) for continuous variables, and as percentages for categorical variables. Kolmogorov-Smirnov test was used to evaluate whether each parameter followed a normal distribution. Analysis of variance (ANOVA) with the Bonferroni post hoc analysis was used to evaluate marker level differences between sample types (control, pretreatment cases and post-treatment cases). Bivariate correlation was performed using the Spearmańs correlation test. OS and PFS were analyzed by means of the Kaplan-Meyer method, and survival curves of subgroups were compared using the log-rank test. All statistical analyses were performed with the SPSS computer software, version 15.0 for Windows (SPSS Inc., Chicago, Ill, USA). Probability values <0.05 were regarded as statistically significant.

## Results

### Patient Characteristics, Treatments and Outcome

Between January 2009 and September 2010, sixty patients and sixty healthy subjects were included. Table 1 summarizes the clinical characteristics of the study group. Treatments received by patients are described in Table 2. The most remarkable treatment-related toxicity was hematological, with 15% of grade 3–4 neutropenic fever, and two deaths from this cause (both patients received cisplatin+docetaxel). Only 2 patients presented with thrombotic events during treatment: one patient with pulmonary thromboembolism, and one patient with deep venous thrombosis (both patients received cisplatin+docetaxel). Two patients presented with severe pulmonary hemorrhage, one of them related to bevacizumab. Regarding efficacy, more than half of patients presented an objective response (58.3%). Median PSF was 10 months (95% CI, 7.9 to 12 months) and median OS was 11 months (95% CI, 8.8 to 13.1 months). Only 36 patients were alive at the time scheduled for the second blood extraction.

**Table 1:** Clinical characteristics of 60 patients with NSCLC and 60 controls.

	Patients		Controls	
	n	%	n	%
Age (years)	61.6±9.7		59,1±8,8	
Sex (males)	54	90	51	85
Smokers	48	80	32	53
Former smokers	8	13.3	0	0
Non-smokers	4	6.7	28	47
PS 0–1	55	91.7	–	–
PS 2 or more	5	8.3		
Histology			–	–
Squamous cell carcinoma	21	35		
Adenocarcinoma	24	40		
Undifferentiated cells	10	16.7		
Large cells	5	8.3		
Clinical stage			–	–
III inoperable	23	39.6		
IV	37	60.3		
Metastasis			–	–
Brain	9	15		
Lung	14	23.3	–	–
Liver	6	10	–	–
Adrenal	12	20	–	–
Bone	17	28.3	–	–
Pleural	4	7	–	–

Age is expressed as mean ± SD.

**Table 2:** Treatment administered to the 60 patients with NSCLC.

Chemotherapy	Patients	%
Cisplatin+docetaxel	32 ^(1)^	53.3
Carboplatin+paclitaxel	12 ^(2)^	20
Cisplatin+pemetrexed	3 ^(3)^	5
Cisplatin+docetaxel+bevacizumab	6	10
Carboplatin+paclitaxel+bevacizumab	2	3.3
Cisplatin+pemetrexed+bevacizumab	4	6.6
Others	1	1.7

(1) Six patients received concomitant radiation therapy (Stage III).

(2) Six patients received concomitant radiation therapy (Stage III).

(3) Three patients received concomitant radiation therapy (Stage III).

### Circulating Marker Levels and Correlation Analysis

Levels of CECs, MPs and ETG in patients and controls are shown in Table 3. The mean levels of CECs and MPs were significantly higher (P>0.0001) in patients compared with controls. However, no significant differences were observed between pre- and posttreatment levels of CECs and MPs. Levels of ETG were increased significantly in patients, only in pretreatment samples (P<0.01).

**Table 3:** Levels of CECs, MPs and ETG in patients with NSCLC pre- and post treatment with chemotherapy and in controls.

	Pre-treatment	Post-treatment	Controls	*P*1	*P*2	*P*3
	(n = 60)	(n = 36)	(n = 60)			
CECs (cells/mL)	101±76	159±11	11±6	0.25	<0.0001	<0.0001
MPs (MP/µL)	6035±5749	4999±4706	1848±2058	0.4	<0.0001	<0.0001
ETG (nMol)	209±86.4	183±71	158±59	0.3	<0.01	0.2

Data are presented as mean±SD. CECs, circulating endothelial cells; MPs, microparticles; ETG,

endogenous thrombin generation. *P*1, pre-treatment *vs* post-treatment; *P*2, pre-treatment *vs* control; *P*3, post-treatment *vs* control.

The correlations between levels of circulating markers showed that MP levels positively correlated with ETG levels, both before (Spearman r = 0.60, P<0.001) and after treatment (Spearman r = 0.68, P<0.001).

### Circulating Markers and Clinical Outcome

**Figure 1:**
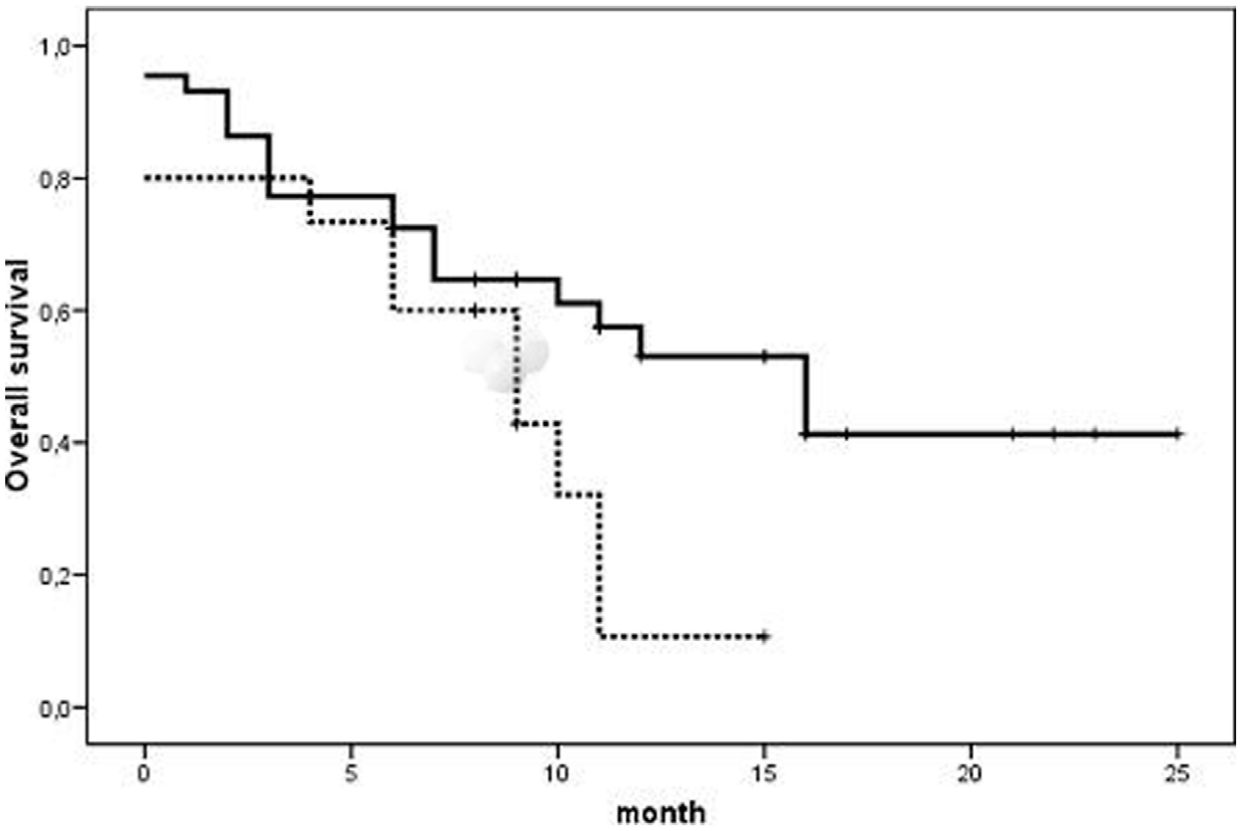
Kaplan-Meyer analysis of survival. The upper reference limit was calculated as values of CECs >152 cells/mL (corresponding to the 75^th^ percentile of the patient group) CECs: circulating endothelial cells.

The analysis of the associations between circulating markers and clinical outcome showed that basal MPs levels >3,278 MP/µL (corresponding to the 99^th^ percentile in controls) were associated with better PFS (15 vs 9 months; p = 0.04) and OS (16 vs 10 months; p = 0.04). No such association was found for CECs or ETG (Table 4). Moreover, basal levels of CECs >152 cells/mL (corresponding to the 75^th^ percentile in patients) were associated with a worse OS (16 months vs 9 months respectively; Log rank 4.3; p = 0.04) (Figure 1).

**Table 4:** Kaplan-Meyer analysis of survival.

	Overall Survival				Progression free survival			
Variables	Median OS	Median OS	Chi-	*p*	Median PFS	Median PFS	Chi-	*p*
	Altered levels	Normal levels	square		Altered levels	Normal Levels	square	
CECs>20 cells/mL	8	11 (8.9–13)	0.52	0.5	9 (6–11)	10 (7.9–12)	0.64	0.4
MPs>3278 MP/µL	16	10 (6.1–13.8)	4.3	0.04	15(11.4–18)	9 (8–12)	4.02	0.04
ETG>275 nMol	7(0.11–13.8)	11 (6.6–15.3)	0.05	0.8	6 (0–14)	11	1.5	0.2

OS and PFS in patients according to pre-treatment marker levels (compared to 99^th^ percentile of controls).The upper reference limit levels of parameters were calculated as values higher than the 99th percentile of the control group (healthy subjects) and were set at: CECs = 20 cells/L; MPs = 3278 MP/µL; ETG = 275 mM. CEC, circulating endothelial cells; MPs, microparticles; ETG, endogenous thrombin generation.

## Discussion

The results of the current study show that the CEC, MP, and ETG levels are elevated in patients with NSCLC in comparison with healthy controls. Chemotherapy did not change the levels of these markers. We have also found an association between high MP levels, both before and after treatment, and survival. Moreover, we have observed an association between pretreatment levels of CECs and survival.

Our results show an association between high pre- and posttreatment levels of MPs and longer survival. To our best knowledge, this is the first study showing such an association in patients with NSCLC. There is growing interest in the characterization of MPs and in their potential role as biomarkers in different diseases [31]. In patients with castration-resistant prostate cancer, high platelet-derived MP levels were associated with a short survival [32]. A study in patients with respiratory distress syndrome showed a positive correlation between high levels of leukocyte-derived MPs and survival [33]. Results from an experimental study with a murine Lewis lung carcinoma model, demonstrated that lymphocyte-derived MPs exerted antiangiogenic and proapoptotic effects that led to the inhibition of tumor growth by reducing VEGF levels [34]. Although we have not characterized MPs by their cellular origin, these findings could provide and explanation for the positive association between MP levels and survival that we have found in the present study.

A strong correlation was observed between MP levels and ETG. This finding is in accordance with previous data, indicating that the presence of MPs in plasma significantly affects ETG in both normal [35] and pathological conditions [23], [36]. The increased number of circulating MPs and their correlation with high ETG levels observed in our study corroborates the role of ETG in cancer hypercoagulability, as previously reported [29]–[33]. Furthermore, as recently demonstrated, the measurement of ETG may identify cancer patients at high risk of venous thromboembolism [37].

We observed an association between high pretreatment levels of CECs and poor survival. There is no conclusive data on the prognostic value of CECs in cancer. Most studies agree that high levels of CECs are associated with a poor prognosis [10], [12]–[13]. However, other studies have provided controversial results [11], [14]. Beerepoot et al. [10] studied the levels of viable CECs in patients with various types of cancer; they found that CEC counts were higher in patients than in healthy subjects, and that chemotherapy increased CEC counts. These authors defined CECs as CD146+, with additional staining with CalceinAM to quantify viable cells; no other markers, such as UEA1, were used to confirm the endothelial nature of the cells. The study by Kawaishi et al. [14] found an association between increased pre-treatment levels of CECs and good prognosis in patients with NSCLC. In this study, the endothelial marker CD146, the DNA marker DAPI, and the activation marker CD105 were used to select CECs, along with CD45 to discard hematopoietic cells. By contrast, in patients with colorectal carcinoma treated with chemotherapy and bevacizumab, Ronzoni et al. [12] found resting CEC counts to be lower in patients who achieved a radiological response in comparison with patients not achieving response. The study by Matsusaka et al. [13], showed that patients with pre-treatment levels of CXCR4-positive CECs >20% of mononuclear cells had a worse PFS and OS. These results are consistent with those obtained in our study, where high levels of CECs were associated with poor OS. Given the variety of techniques used for quantification of endothelial cells, efforts are needed to identify the most appropriate cellular phenotypes for each clinical situation and to explain current controversies.

Our findings provide new data on the prognostic value of MPs and CECs in patients with NSCLC. The role of MPs and CECs as potential biomarkers in patients with NSCLC, treated with conventional chemotherapy or targeted therapies, warrant further study, since a better understanding of their role can open new perspectives in the fields of diagnosis and treatment.
